# Meteorological Register

**Published:** 1834-04-01

**Authors:** 


### METEOROLOGICAL REGISTER,

FROM DECEMBER 1 TO FEBRUARY 28.

By Messrs. HARRIS and Co., Mathematical Instrument Makers, 50, High Holborn.

| | Thermometer. | | Barometer. | | De Luc's Hygrometer. | | Winds. | | Atmospheric Variations. |
|---|---|---|---|---|---|---|---|---|---|
| 1833. | max. | min. | max. | min. | max. | min. | | | |
| Dec. 1 to 7 | 56 | 41 | 29.91 | 29.29 | 82 | 75 | W | WNW | fine, cloudy, sleet |
| 14 | 56 | 34 | .85 | .10 | 83 | 76 | NW | WNW | foggy, fine, rain |
| 21 | 55 | 38 | .90 | .21 | 81 | 75 | W | WSW | fine, rain, cloudy |
| 1834. 30 | 55 | 35 | .90 | .15 | 89 | 75 | SW | WSW | showery, rain, fine |
| Jan. 1 to 7 | 51 | 37 | 30.03 | .31 | 80 | 75 | NNW | SW | cloudy, showery, rain |
| 14 | 51 | 38 | 29.45 | 28.90 | 85 | 80 | SW | SSW | fine, rain, cloudy |
| 21 | 53 | 37 | .80 | 29.16 | 83 | 78 | SW | WSW | rain, cloudy, fine |
| 31 | 57 | 34 | 30.15 | .22 | 85 | 73 | WSW | WNW | fine, rain, cloudy |
| Feb. 1 to 7 | 50 | 32 | 29.92 | .80 | 82 | 75 | SSW | W | foggy, cloudy, fine |
| 14 | 46 | 31 | 30.20 | .57 | 80 | 78 | W | SW | foggy, rain, fine |
| 21 | 50 | 32 | .16 | .75 | 79 | 72 | WNW | WSW | fine, foggy, cloudy. |
| 28 | 55 | 38 | .25 | .63 | 80 | 75 | WSW | SW | |

The quantity of Rain fallen in December, 2 inches and 60-100ths.
— — — January, — 98-100ths.
— — — February, — 27-100ths.

